# HIV-1 RT Inhibitors with a Novel Mechanism of Action: NNRTIs that Compete with the Nucleotide Substrate

**DOI:** 10.3390/v2040880

**Published:** 2010-03-30

**Authors:** Giovanni Maga, Marco Radi, Marie-Aline Gerard, Maurizio Botta, Eric Ennifar

**Affiliations:** 1 Istituto di Genetica Molecolare, IGM-CNR, Via Abiategrasso 207, 27100 Pavia, Italy; E-Mail: maga@igm.cnr.it (G.M.); 2 Dipartimento Farmaco Chimico Tecnologico, University of Siena, Via Alcide de Gasperi 2, 53100 Siena, Italy; E-Mails: marcoradi@gmail.com (M.R.); botta.maurizio@gmail.com (M.B.); 3 Architecture et Réactivité des ARN, Université de Strasbourg, CNRS, 15 rue René Descartes, F-67084 Strasbourg, France; E-Mail: ma.gerard@ibmc.u-strasbg.fr (M.-A.G.)

**Keywords:** HIV, reverse transcriptase, competitive inhibitors

## Abstract

HIV-1 reverse transcriptase (RT) inhibitors currently used in antiretroviral therapy can be divided into two classes: (i) nucleoside analog RT inhibitors (NRTIs), which compete with natural nucleoside substrates and act as terminators of proviral DNA synthesis, and (ii) non-nucleoside RT inhibitors (NNRTIs), which bind to a hydrophobic pocket close to the RT active site. In spite of the efficiency of NRTIs and NNRTIs, the rapid emergence of multidrug-resistant mutations requires the development of new RT inhibitors with an alternative mechanism of action. Recently, several studies reported the discovery of novel non-nucleoside inhibitors with a distinct mechanism of action. Unlike classical NNRTIs, they compete with the nucleotide substrate, thus forming a new class of RT inhibitors: nucleotide-competing RT inhibitors (NcRTIs). In this review, we discuss current progress in the understanding of the peculiar behavior of these compounds.

## 1. Introduction

Current treatments against human immunodeficiency virus type 1 (HIV-1) infections include six different classes of drugs, targeting the three viral enzymes (more than 20 different compounds in clinical use target the protease, reverse transcriptase and integrase), the virus fusion process (enfuvirtide/T-20 targets the viral gp41) or viral entry (maraviroc targets the human CCR5 cellular coreceptors) [1]. Combinations of these drugs are used in a treatment strategy known as HAART (highly active antiretroviral therapy). The viral reverse transcriptase (RT) was identified early as an interesting target, with the use of zidovudine (AZT) [2] approved for the treatment of HIV-1 infections in 1987. In spite of more than 20 years of developments, RT remains an important target in antiretroviral therapy, since 12 out of 25 individual agents licensed for the treatment of HIV-1 infection target its polymerization active site.

Currently, approved RT inhibitors are divided into two classes: eight nucleoside and nucleotide RT inhibitors (NRTIs), and four non-nucleoside RT inhibitors (NNRTIs). NRTIs are phosphorylated by cellular enzymes and converted into their active NRTI triphosphate form. Once activated, they compete with the natural nucleoside triphosphate (dNTP) for binding the RT polymerase active site, and after their incorporation into the primer strand, act as terminator of DNA synthesis due to the lack of a 3′-hydroxyl group [3]. In contrast, NNRTIs comprise a structurally diverse family of compounds all binding the same hydrophobic pocket (non-nucleoside inhibitor binding pocket, NNIBP) located in the palm domain of the p66 subunit and about 10 Å from the polymerase catalytic site [4–7]. Upon binding the HIV-1 RT, NNRTIs do not interfere with dNTP binding, but rather lead to unproductive complexes by altering the conformation or mobility of RT, thereby exerting a non-competitive inhibition [8–10]. In addition, whereas NRTIs are characterized by a rather large antiviral spectrum on several *Lentiviridae* species, NNRTIs are highly specific for HIV-1 RT and result in less adverse effects than NRTIs.

**Figure 1.**
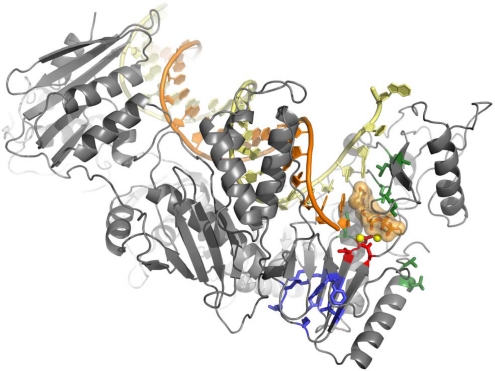
Localization of more frequent NNRTI- and NRTI-associated resistance mutations on the RT/primer-template complex [17]. NNRTI-selected mutations (in blue) are all localized in the NNIBP, whereas NRTI-selected mutations (in green) are distributed around the dNTP binding site. The template strand is shown in yellow, while the growing complementary DNA chain is in orange. The catalytic D110, D185 and D186 are shown in red, the two magnesium cations required for the catalytic reaction as yellow spheres, and the incoming nucleotide is represented in space filling mode.

New strategies to inhibit RT enzymatic activities and to overcome viral resistance are still under investigation. A successful example is illustrated by the recent development of the very promising “next-generation” NNRTIs developed by Tibotec, namely etravirine (TMC125, ETV) [11,12] that has been approved for HIV-1 treatment in 2008, and the rilpivirine (TMC278) [13,14] that is currently in Phase III clinical trials [15,16]. However, long-term treatment of HIV-1 by antiretrovirals is prevented by incomplete viral suppression resulting from the rapid emergence of drug-resistant mutants. Since NRTIs and NNRTIs target different binding sites and are using distinct inhibition mechanisms, the mechanism of resistance is specific for each class of inhibitor and leads to the selection of completely different sets of resistance mutations: whereas NRTI-associated mutations have a rather broad spatial distribution in the neighborhood of the nucleotide substrate binding site, NNRTI-resistance mutations are concentrated in the NNIBP (Figure 1).

NNRTIs have become a cornerstone of HAART. However, in spite of the remarkable potency of currently marketed NNRTIs, the rapid selection of single mutations can confer resistance to most NNRTIs, leading to an almost complete loss of their activity, without significantly affecting the viral infectivity. Consequently, there is an urgent need for the development of new RT inhibitors with an alternative mechanism of action and exhibiting different resistance profiles.

For this purpose, several RNaseH inhibitors targeting the two metal ions essential for this RT activity have already been developed [18–21]. These compounds are, however, so far lacking antiviral activity. In 2006, the indolopyridones were discovered almost concurrently by two groups [22,23] and led to the identification of a new class of RT inhibitors targeting the polymerization activity of the RT, but with a mechanism of action that involves a competitive binding with the incoming dNTP. Only a few months later, another family of non-nucleoside compounds, the 4-dimethylamino-6-vinylpyrimidines (DAVPs), was reported to inhibit the RT polymerase activity also by a competitive mechanism with the nucleotide substrate and distinct from those of common NNRTIs [24]. Because their original mechanism of action is competitive with the nucleotide substrate - although they are not chemically-related to NRTIs - it was proposed to refer to members of this class of compounds as “nucleotide-competing RT inhibitors” (NcRTIs) [25]. This review focuses on progress over the past three years in understanding the mechanism of action of this new family of RT inhibitors.

## 2. Indolopyridones (INDOPYs)

**Figure 2.**
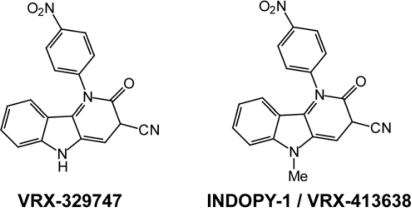
Chemical structures of indolopyridones.

The indolopyridones VRX-329747 {1-(4-nitrophenyl)-2-oxo-2,5-dihydro-1H-pyrido[3,2-b]indole-3-carbonitrile} and VRX-413638 (or INDOPY-1) {5-methyl-1-(4-nitrophenyl)-2-oxo-2,5-dihydro-1H-pyrido[3,2-b]indole-3-carbonitrile} were identified during cell-based high-throughput screenings of Valeant and Tibotec compound libraries aimed at identifying new HIV-1 inhibitors that were effective against drug-resistant mutants, possibly with a new mechanism of action [22, 23]. These two compounds emerged as potent HIV-1 inhibitors (EC_50_ = 150–200 nM for VRX-329747 and EC_50_ = 20–30 nM for INDOPY-1) with a low cellular toxicity (CC_50_ > 100 μM). *In vitro* enzymatic experiments based on inhibition of DNA synthesis led to the conclusion that both compounds were targeting the reverse transcription step of HIV-1 replication. In spite of their non-nucleosidic chemical structure (Figure 2), several features of indolopyridones (INDOPYs) were clearly inconsistent with their classification as NNRTIs, thus calling for more investigations of their mechanism of action.

A first striking feature of INDOPYs is their antiviral spectrum, which is clearly distinct from that of NNRTIs and NRTIs. Whereas NNRTIs are highly specific for HIV-1, INDOPY-1 remains active on HIV-2 (EC_50_ = 180 nM on HIV-2 ROD) and SIV (EC_50_ = 210 nM on SIV Mac251) [22]. However, the antiviral activity of INDOPY-1 seems restricted to lentiviruses since no inhibition was reported on other retroviruses like Moloney murine sarcoma virus (Mo-MSV) [22], thus contrasting with the broad antiretroviral spectrum of NRTIs that do not markedly discriminate between RTs from different origins and even recognize the DNA polymerase of human hepatitis B virus.

**Figure 3.**
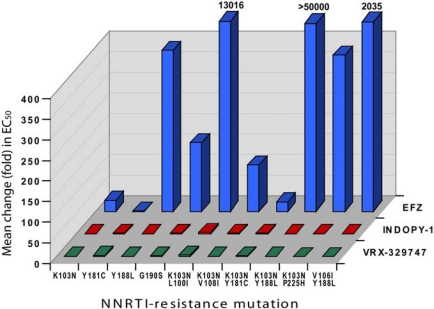
Activity profile of the indolopyridones VRX-329747 (green) and INDOPY-1 (red) compared to efavirenz (blue) on HIV-1 molecular clones carrying NNRTI-selected mutations. The mean fold change is relative to the wild-type EC_50_. Adapted from [23].

Another important feature of INDOPYs is their unique activity profile towards drug-resistant RT mutants. Antiviral tests conducted on HIV-1 molecular clones revealed that VRX-329747 and INDOPY-1 are not, or are only moderately, affected by several representative NNRTI-selected mutations, with a maximum of ∼4-fold decrease of the EC_50_ towards the double K103N-L100I RT mutant [23]. This behavior contrasts with that of efavirenz (EFZ), which is dramatically affected by these mutations (Figure 3), with a complete loss of its antiviral activity (more than 100-fold change) for most of the tested mutants.

In addition to this high activity on NNRTI-resistant strains, INDOPYs also revealed an unusual activity profile against NRTI-associated mutations. While INDOPY-1 is not affected by thymidine analog resistance mutations (TAMs, including M41L/L210W/T215Y and D67N/K70R/T215F/K219Q mutations [26–28]) or other frequent NRTI-induced mutations, its activity is moderately reduced by M184V or Y115F mutations (3- to 8-fold reduced activity) and strongly reduced by the combination of these two mutations (more than 100-fold change) [22,23]. On the contrary, hypersusceptibility was observed for viruses carrying the K65R mutation (2- to 5-fold increased activity) [22,23]. Noticeably, the K65R mutation causes reduced efficiency of all NRTIs, with the exception of zidovudine (AZT) [29].

Regarding mutations selected by viruses cultured in the presence of INDOPYs, it was found that four NRTI-resistance mutations were selected after six months of VRX-329747 selection [23]: M184V, a well-characterized mutation associated with a strong resistance to lamivudine (3TC) and emtricitabine (FTC); M41L, which strongly reduces susceptibility to stavudine (D4T) and AZT; A62V and V118I, two accessory mutations occurring with TAMs, the Q151M multi-dideoxynucleoside-resistance mutation or the 69 insertions [30–32] and associated with subtle reductions in susceptibility to multiple NRTIs. Together with these four NRTI-associated mutations, two new mutations were found: S68N and G112S. All these mutations are located around the incoming nucleotide binding site, which is consistent with a new mechanism of action for VRX-329747 compared to NNRTIs.

In view of this remarkable resistance profile, *in vitro* experiments have been carried out on INDOPY-1 to provide more insight into its mode of inhibition of proviral DNA synthesis [22]. Detailed analysis of the DNA polymerization products in the presence of INDOPY-1 showed that the inhibitory mechanism was not affected by the identity of the incoming nucleotide, being competitive towards all four dNTPs. Moreover, these studies revealed that the inhibition was not resulting from chain termination, as it is the case for NRTIs, and was reversible. However, equilibrium-binding experiments showed that INDOPY-1 was able to bind and stabilize the binary complex of RT with its DNA template.

Interestingly, the binding of INDOPY-1 was affected by the identity of the last nucleotide at the 3′-end of the primer strand, with T and C being the most efficient in promoting INDOPY-1 binding. These data clearly indicated that INDOPY-1 preferentially binds to RT after the incorporation of pyrimidines. Similarly to all RNA and DNA polymerases, HIV-1 RT also undergoes a translocation step after nucleotide incorporation, exposing the next template position to its active site ready to accept the incoming nucleotide. Site-specific footprinting showed that INDOPY-1 binds to HIV-1 RT in its post-translocation conformation, in place of a nucleotide, and then induces a stable dead-end complex. Thus, the inhibition of HIV-1 RT by INDOPY-1 is due to a “freezed” conformation at the post-translocation step, which prevents nucleotide binding [22]. In order to resume synthesis, INDOPY-1 must dissociate from RT, an event which eventually occurs, albeit with slow kinetics.

**Figure 4.**
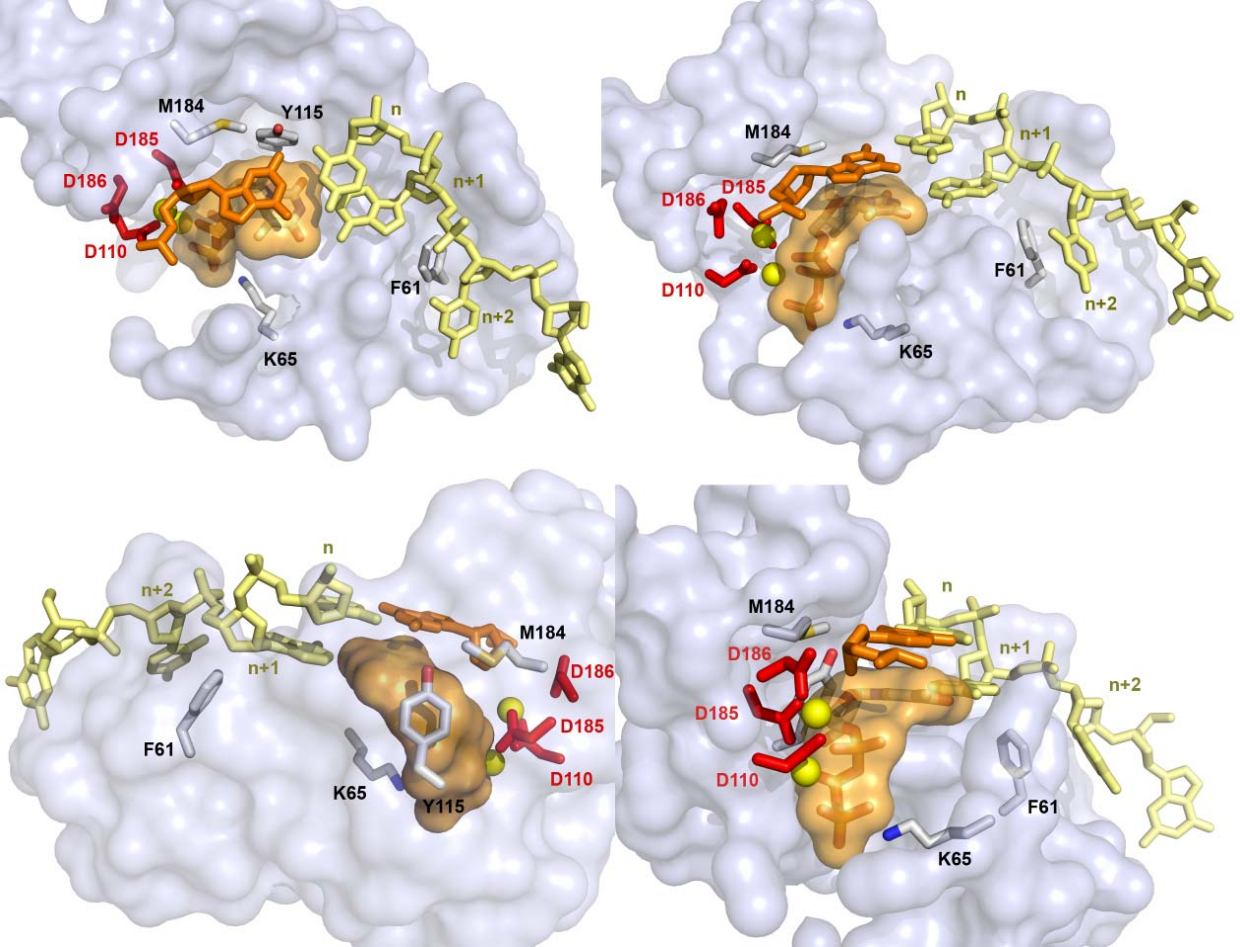
Four different views showing the local environment around the HIV-1 RT polymerase active site and the putative INDOPY-1 binding site. The structure of the trapped RT/primer-template/incoming dNTP catalytic complex [17] is represented. The three catalytic aspartic acids are shown as red sticks, the two required magnesium ions as yellow spheres, the DNA template in light yellow (positions *n*, *n* + 1 and *n* + 2 are labeled) and the DNA primer in orange. Residues affecting the inhibitory effect of INDOPY-1 (F61, K65, Y115 and M184) and the incoming dTTP are also shown as sticks. The solvent accessible surface of the dTTP is shown in orange transparency. Adapted from [25].

To investigate the correlation between phenotypic susceptibility to INDOPY-1 and the enzymatic properties of RT, a detailed analysis on mutants affecting the activity of the inhibitor in cell-based assays was also performed *in vitro* [25] (Figure 4). These studies revealed that mutations M184V and Y115F both induced a 2-fold increase of IC_50_ values for INDOPY-1, with a reduction of the enzyme pausing. A cumulative effect was observed on the double M184V/Y115F mutant, with a >15-fold increase in IC_50_. The synergic effect of these two mutations might be attributed to differences in inhibitor binding: whereas the M184V mutation reduces INDOPY-1 affinity (∼6-fold change), the Y115F mutation mostly results in an increased binding of the natural nucleotide substrate (∼3-fold increase in affinity). However, none of these mutations affect the aforementioned sequence specificity of INDOPY-1. On the contrary to M184V and Y115F mutations, the inhibitory effect of INDOPY-1 was slightly increased in presence of the K65R mutation that reduces the affinity of the nucleotide substrate (∼2-fold change) and increases the INDOPY-1 binding (1.6-fold change). This likely accounts for the observed hypersusceptibility of this RT mutant towards INDOPY-1. Taken together, these results are therefore in good agreement with cell-based assays [22]. Binding of INDOPY-1 was also investigated *in vitro* on the F61A mutant. This replication-defective RT mutant is characterized by an increased fidelity corroborated by a reduction in sensitivity to dideoxy-thymidine analogs and shows defects in nucleotide binding, but not in primer-template binding [33–35]. It was shown that the F61A mutation strongly enhances the IC_50_ value (>40-fold change) of INDOPY-1. In addition, whereas no stable ternary complex could be observed with the natural nucleotide substrate, a stable F61A RT/primer-template/INDOPY-1 complex can be formed [25], thus indicating that a decrease of the nucleotide binding can be sufficient to increase the INDOPY-1 inhibitory effect.

Finally, the latter study also examined the effect on INDOPY-1 binding of abasic sites in the template strand at positions opposite the 3′-end of the primer (*n*) and the incoming nucleotide substrate (*n* + 1) (Figure 4). It was found that an abasic site at position *n* completely abolishes binding of INDOPY-1, whereas a modification of the n + 1 position did not significantly affect the binding [25]. This suggests that interactions with the 3′-end of the primer - likely stacking interactions with the nucleotide base - are a prerequisite for INDOPY-1 binding, while interactions with the nucleotide opposite the incoming dNTP – presumably hydrogen bonds with the base – are dispensable. These findings also well correlate with the marked preference of INDOPY-1 for pyrimidines residue at the 3′-end of the primer, which also suggests a predominant role of stacking interactions with this position.

## 3. 4-Dimethylamino-6-vinylpyrimidines (DAVPs)

**Figure 5.**
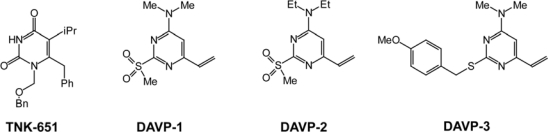
Chemical structures of DAVP derivatives and of the reference compound TNK-651.

Independently from INDOPYs, the development of a straightforward combinatorial approach for the synthesis of 6-vinylpyrimidine derivatives [36] led to the discovery of a new family of RT inhibitors, the 4-dimethylamino-6-vinylpyrimidines (DAVPs, Figure 5) [24,37]. Because DAVPs are structurally related to the non-nucleoside RT inhibitor TNK-651 [38], an analog of the 1-[(2-hydroxyethoxy)methyl]-6-(phenylthio)thymine (HEPT) family of NNRTIs (Figure 5), it was initially expected that they would also behave as NNRTIs. However, subsequent enzymological studies performed on DAVPs 1–3 revealed a competitive inhibition mechanism with the incoming nucleotide substrate reminiscent of those of INDOPYs and consistent with the classification of these compounds as NcRTIs [24,37].

**Figure 6.**
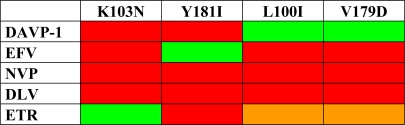
Comparative resistance profiles of Efavirenz (EFZ), Nevirapine (NVP), Delavirdine (DLV), Etravirine (ETR) and DAVP-1 towards four NNRTI-associated RT mutants. Red squares stand for strong loss of activity (>5-fold change), orange for intermediate loss of activity and green for weak loss of activity (<2-fold change).

Out of these three compounds, DAVP-1 emerged as the most active one on the wild-type enzyme (K*_i_* = 8 nM) in cell-free assays [24]. However, whereas no significant loss of activity was observed *in vitro* on L100I or V179D (K*_i_* = 12 and 15 nM, respectively) mutants, the affinity of the compound is severely affected by K103N and Y181I mutations (K*_i_* = 3.2 and 39 μM, respectively). This activity profile thus differs from that of INDOPYs, which are unaffected by these mutations, and from licensed NNRTIs (Figure 6) since L100I and V179D mutations both strongly affect the inhibitory effect of efavirenz, nevirapine (NVP) and delavirdine (DLV) *in vitro* [39–41] and in cell-based assays [42]. In addition, these two mutations belong to the etravirine (ETV) resistance-associated mutations [43,44], causing moderate resistance to this drug [42, 45].

In view of this unusual resistance profile and to determine their mechanism of inhibition, *in vitro* reverse transcriptase titrations assays have been carried out on DAVPs in presence of increasing concentrations of either a RNA/DNA nucleic acid or the nucleotide substrates. These experiments revealed that the inhibition of the HIV-1 RT by DAVPs was sensitive to the nucleotide concentration, resulting in an increase in the apparent Km for dTTP, but not to the nucleic acid concentration [24]. The reference compound TNK-651 used as a comparison showed a typical non-competitive mechanism with respect to the nucleotide substrate (no significant variation of Km value for dTTP), indicating that the inhibitor binds independently from the natural substrate, as expected for a NNRTI.

Further insight into the mechanism of DAVP binding to the HIV-1 RT was provided by steady-state kinetic enzymatic studies performed with DAVP-1 on various RT/substrate complexes: the free enzyme, the RT/primer-template binary complex and the RT/primer-template/incoming nucleotide ternary complex (Table 1). These experiments showed that DAVP-1 is able to bind with the same affinity (K*_i_* = 8 nM) to the unliganded RT and the RT/primer-template binary complex. However, although the equilibrium dissociation constant (K*_i_* = k_off_/k_on_) are equivalent, a 13-fold faster association and 8-fold slower dissociation rate for the binary RT-primer/template complex are obtained with respect to the free RT [37]. This is in contrast with INDOPY-1, which is highly specific for the RT/primer-template complex since no binding was detected to the unliganded RT [23]. Regarding the catalytically competent ternary complex with both the primer-template and the nucleotide substrate, a 140-fold decrease of the association rate relating to the binary complex was observed, but no significant change in the dissociation rate [37]. As a result from these kinetic studies, it therefore appears that the binding of DAVP-1 to the free enzyme or to the RT/primer-template binary complex is more stable than to the RT/primer-template/dNTP complex. This peculiar behavior excludes a classical competitive inhibition mechanism for DAVPs, resulting only from a binding of the inhibitor to the polymerase catalytic site of the binary complex, as for NRTIs. Finally, although DAVP-1 was shown to be highly active in cell-free assays, its antiviral efficiency remains to be tested in cell-based assays.

## 4. Structural Biology Studies of NcRTIs

Structural biology studies are currently in progress for providing a detailed molecular view of how NcRTIs are interacting with the HIV-1 RT. X-ray crystallography already contributed precious and highly detailed information of NNRTIs bound to the free RT (see for instance [5, 6, 7]), thus helping for the development of the second generation of this class of inhibitors [13, 46]. However, at variance with NNRTIs that were successfully co-crystallized with the free RT, attempts to co-crystallize INDOPY-1 with the unliganded RT failed [23], likely because it is highly specific for the binary RT/primer-template complex. Unlike INDOPY-1, DAVPs bind the unliganded RT in addition to the binary complex and DAVP-1 was successfully co-crystallized with the RT in absence of nucleic acids [47]. Obtained crystals revealed a novel crystal packing differing from previously-reported RT structures.

**Figure 7.**
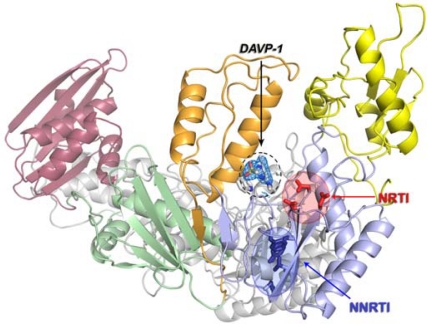
Overall view of the HIV-1 RT/DAVP-1 complex showing the relative position of binding sites for NNRTI (circled in blue, important residues of the NNIBP are shown as blue sticks) and NRTI (circled in red, the three catalytic D110, D185 and D186 residues are represented as red sticks). The p51 subunit is represented in grey whereas p66 subdomains are color-coded: fingers in yellow, thumb in orange, palm in light blue, connection in green and RNAseH in purple. The electron density map at 2.8 Å resolution is represented around DAVP-1. Residue W266 is also shown as sticks. Adapted from [47].

**Figure 8.**
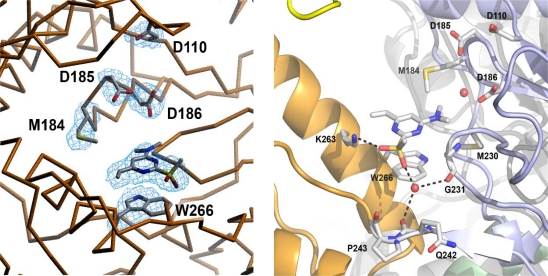
Left: Detailed view of the HIV-1 RT/DAVP-1 complex showing the electron density map at 2.8 Å resolution (in blue) around DAVP-1 and residues D110, M184, D185, D186 and W266. Right: Detailed view of the inhibitor binding pocket. Water molecules are shown as red spheres. Hydrogen bonds are represented with black dotted lines. Adapted from [47].

The structure of the wild-type HIV-1 RT/DAVP-1 complex revealed that the inhibitor does not bind the NNIBP, but rather at a novel site located in a hinge region at the interface between the p66 thumb and p66 palm subdomains, near the polymerase active site (Figure 7 and 8). Most of the residues interacting with the drug belong to an extremely conserved region of the HIV-1 RT from naïve and drug-treated patients [48], suggesting that amino acids forming the DAVP-1 binding site are not prone to mutations. In addition, the drug binding site includes several essential residues for the enzyme activity (Figure 8, right). Firstly, M230 and G231 are two critical residues that belong to the DNA primer grip [49], a highly conserved structural motif [50] comprising the p66 β12-β13 hairpin in HIV-1 RT [51] that helps the positioning of the 3′-hydroxyl end of the primer strand in the polymerase active site (and also involved through residues F227, W229, L234 into NNRTI binding [52]). Secondly, G262, K263 and W266 are located in the core of the αH helix within the “helix clamp”, a motif involved into the primer-template recognition [53]. These residues belong to the minor groove binding track (MGBT) and play an essential role in nucleic acid binding by acting as “protein sensors” of the DNA minor groove [54–56]. Finally, M184 and D186, which are lying within 3.5–4.0 Å from the diethylamino group of DAVP-1, are part of the catalytically crucial YXDD motif (Y_183_MDD_186_ in HIV-1 RT). This motif is highly conserved among retroviruses [57, 58]: residue D186 is one of the three catalytic aspartic acids binding the two divalent magnesium cations required for the polymerization reaction [59], whereas M184 is likely involved into the fidelity of DNA synthesis [60] through interactions with the 3′-end of the primer and the incoming dNTP [17]. This latter residue is the only one, among those contacting DAVP-1, which has been reported to mutate as a result of antiviral drug pressure since the M184V/I substitution is responsible for high level resistance towards the NRTI lamivudine (3TC).

**Figure 9.**
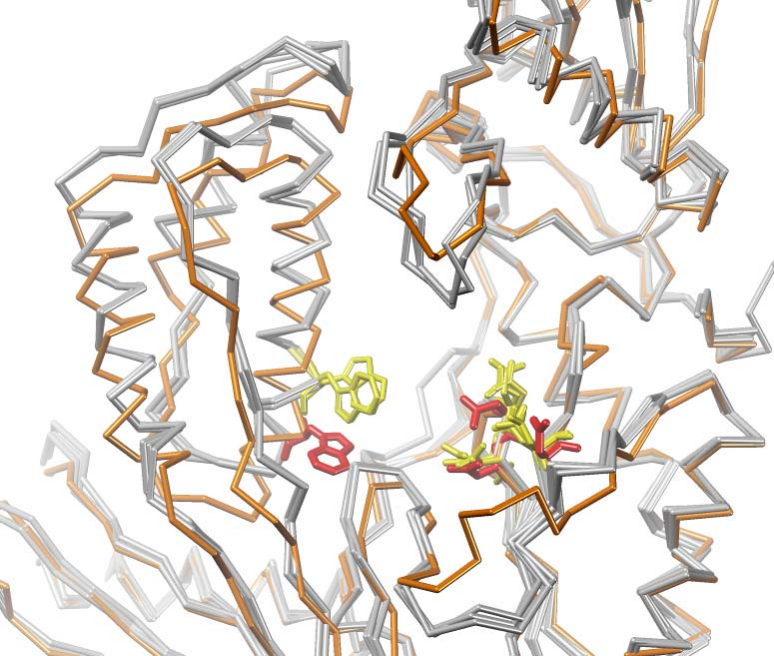
Detailed view of the DAVP-1 binding pocket showing a superposition of the cα backbone in unliganded HIV-1 RT structures (in grey, PDB 1DLO, 1QE1 and 1HQE) and in RT bound to DAVP-1 (in orange, PDB 3ITH and 3ISN). Residues W266, D110, D185 and D186 are shown in yellow in unliganded RT structures and in red in the RT/DAVP-1 complex.

Another feature of the HIV-1 RT/DAVP-1 complex is the conformation adopted by the protein upon drug binding. In RT/NNRTI complexes, the RT undergoes a large structural rearrangement and adopts conformation similar to the one observed when the RT is bound to a DNA primer-template (see [61] for a complete review). In the RT/DAVP-1 complex on the contrary, the p66 thumb subdomain is folded into the DNA-binding cleft, similarly to the conformation reported in unliganded wild-type or drug-resistant RTs [62–64], or in drug-resistant RT bound to ATP [65] (high-resolution structures of unliganded RTs obtained after removal of inhibitor from RT/NNRTI co-crystals [4,66] were not used for comparison since the conformation of the protein is “frozen” by the crystal packing). However, some slight differences are observed compared to the latter structures: a ∼3.0 Å translation of the whole p66 thumb subdomain towards the p66 palm subdomain follows the DAVP-1 binding (Figure 9). The movement is likely initiated by a steric hindrance between the inhibitor and the β12-β13 hairpin (M230 and G231) and is propagated to the p66 thumb subdomain.

This crystal structure therefore reveals that the binding site of DAVP-1 is located in a region critical for the correct positioning of the 3′-OH primer for the in-line nucleophilic attack by the incoming dNTP and the subsequent chemical bond formation with its α-phosphate. It is known that mutations of residues involved in the stabilization of the primer/template junction can reduce the binding affinity for the incoming dNTP, as is the case for the L74 residue, which contacts the template at position *n* + 1 and whose substitution with a valine is responsible for NRTIs resistance [67,68]. Thus, it is likely that the DAVP-1 binding causes a misplacement of the primer, so that the ternary complex formed with the incoming dNTP does not undergo to the “open-to-close” conformational transition required to stabilize the dNTP within the active site, resulting in lack of nucleotide incorporation. This mechanism, which therefore differs from the putative mechanism of action of INDOPYs, is also consistent with the kinetic data, suggesting that in the ternary complex conformation (*i.e.*, in the presence of a stably bound dNTP), binding of DAVP-1 is unfavored [24]. The peculiar resistance profile observed for DAVP-1 suggests that it gains access to its novel binding site not through the same route of the incoming dNTPs, but using structural elements in common with the NNIBP [47]. In this respect, it has to be mentioned that the Y181 residue is located on the same structural element of the catalytic D185 and D186 and that its mutation (as in the case of the Y181I substitution) can induce resistance to NRTIs [69].

This RT/DAVP-1 complex, obtained without nucleic acids, obviously does not completely clarify the competitive mechanism of inhibition of DAVPs, but provides first structural requirements for binding of DAVP-1 to the free RT and for understanding the original inhibition mechanism of this compound. Consequently, ongoing research in our group includes structural studies of a challenging RT/primer-template/NcRTI ternary complex, which would provide more valuable information about NcRTI binding and mechanism of inhibition. To prevent the dissociation of the RT/DNA complex and to trap a covalent stable and homogenous complex, a disulfide cross-linking strategy that has already been successfully used in several X-ray studies [17,70–73] is used for co-crystallization of RT/primer-template complex with DAVP-1 or INDOPY-1 (Gerard *et al.*, unpublished results).

## 5. Conclusions

**Figure 10.**
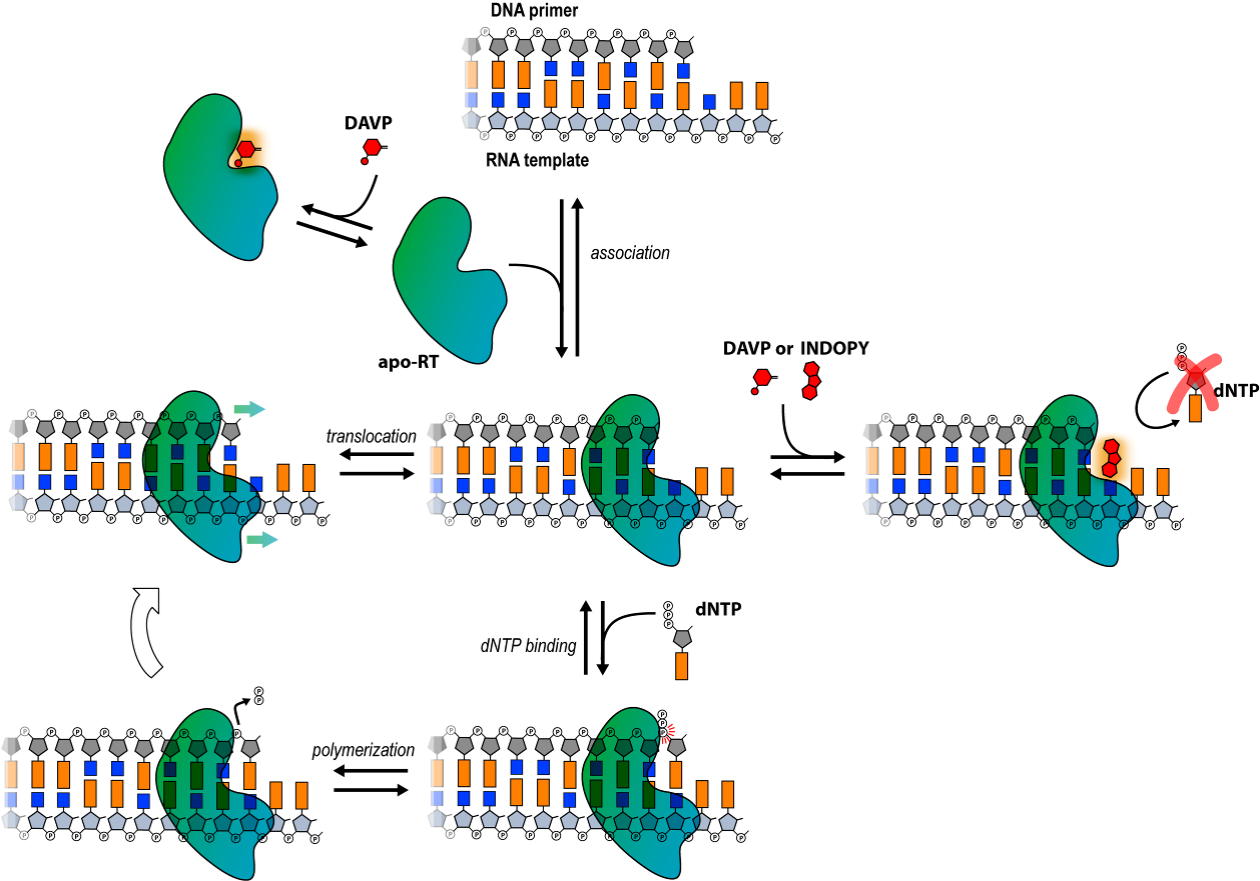
Proposed model for the mechanism of action for DAVPs and INDOPYs. Unlike INDOPYs that are highly specific for the RT/primer-template complex, DAVPs also bind unliganded RT, suggesting some differences in the inhibition mechanism of both families of compounds.

The development of INDOPYs and DAVPs led to the identification of “Nucleotide-competing RT Inhibitors” (NcRTIs), a new class of RT Inhibitors endowed with a novel mechanism of action. Although they are structurally distinct from NRTIs, NcRTIs also bind close to the RT polymerase active site (Figure 4) and compete with the natural nucleotide substrate (Figure 10). However, because NcRTIs (especially INDOPYs) exhibit a resistance profile distinct from NNRTIs and NRTIs - with the exception of the M184V mutation that dramatically affects FTC and 3TC susceptibility, but only moderately INDOPY-1 - they can be potentially used both in naïve and in treatment-experienced patients or combined with classic RT inhibitors, accounting for more exploration of this new class of inhibitors. Further ongoing developments in structural biology include the structure determination of NcRTIs in complex with the RT bound to a DNA primer-template complex, which is clearly required for a better understanding of their mechanism of action and for helping in the design of improved compounds. Finally, the discovery of NcRTIs nicely illustrates that, in spite of more than 20 years of continuous development, research efforts still leads to the development of original RT inhibitors.

## Figures and Tables

**Table 1.** Kinetics of binding of DAVP-1 to different wild-type RT-substrate complexes (adapted from [37]).

	K*_i_* (nM)	k_on_ (M^−1^ s^−1^)^a^	k_off_ (M^−1^ s^−1^)^b^
Free RT	8	1.04 × 10^4^	8.4 × 10^−5^
RT-primer/template	8	14 × 10^4^	1.1 × 10^−5^
RT-primer/template-dNTP	16	0.1 × 10^4^	1.6 × 10^−5^

a Calculated from k_app_ = k_on_•(K*_i_* + [I]);

b Calculated from k_off_ = K*_i_*•k_on_
